# Risk of human papillomavirus infection and cervical intraepithelial lesions in Chinese renal transplant recipients

**DOI:** 10.3389/fonc.2022.905548

**Published:** 2022-07-25

**Authors:** Ming Chen, Qiulin Cui, Meilian Chen, Meng Xia, Duo Liu, Peisong Chen, Changxi Wang, Mian He

**Affiliations:** ^1^ Department of Gynecology, The First Affiliated Hospital, Sun Yat-Sen University, Guangzhou, China; ^2^ Department of Clinical Laboratory, The First Affiliated Hospital, Sun Yat-Sen University, Guangzhou, China; ^3^ Organ Transplant Center, The First Affiliated Hospital, Sun Yat-Sen University, Guangzhou, China

**Keywords:** human papillomavirus, cervical intraepithelial lesions, prevalence, renal transplant recipients, risk

## Abstract

**Objective:**

While human papillomavirus (HPV) infection in women is associated with cervical intraepithelial neoplasia and cervical cancer, HPV testing is not often performed in routine practice for renal transplantation patients. The genotype-specific prevalence of HPV and risk factors for HPV infection are still unclear.

**Methods:**

From 2010 to 2020, patients receiving renal transplantation surgery (referred to as RTRs), who had been screened for HPV infection one year after transplantation were enrolled. A comparison cohort of four age- and marital status-matched healthy individuals was selected for RTRs. The clinical characteristics and cervical screening results of RTRs were analyzed.

**Results:**

Our study included 196 female renal transplant recipients (RTRs), none of whom had been vaccinated against HPV. Overall high-risk HPV (hrHPV) infection and abnormal cytology rates in the RTR group were 23.5% and 20.9%, respectively. The odds ratios of hrHPV infection and cervical intraepithelial neoplasia grade 2+ in RTRs vs. non-RTRs were 3.033 (95% CI, 2.013-4.568) and 3.628 (95% CI, 1.863-7.067), respectively. The prevalence of HPV16 in RTRs was much higher (30.4% vs. 8.3%, P=0.002). The multi-infection rate was much higher in HPV-infected RTRs (23.9% vs. 1.14%, P<0.001). The only risk factor for hrHPV infection was the duration of immunosuppression, which increased with time.

**Conclusion:**

RTRs had significantly higher HPV infection rates and increased risks of HPV-related cervical premalignancies and cancers due to the immunosuppressed state. The duration of immunosuppression is a risk factor for transplant recipients. Female RTRs may benefit from more frequent cervical cancer screening after renal transplantation than healthy women. Prospective research on HPV infection dynamics in RTRs and optimal screening methods should be further explored in the future.

## Introduction

The number of renal transplantation recipients and their life expectancy are increasing. There were 229,887 patients with a functioning kidney transplant in the United States in 2018 (1). In China, there were 7224 kidney transplantations from deceased donations and 1795 from living relative donations in 2016, according to data from the Chinese Scientific Registry of Kidney Transplantation (CSRKT) (2) To prevent graft rejection, renal transplant recipients (RTRs) receive lifelong immunosuppressive treatment (3). Transplant recipients are living longer on immunosuppressive therapy and therefore are exposed to an increased risk of infections and cancer (4). Cervical cancer is one of the most common gynecological cancers in the world. Persistent infection with high-risk human papillomavirus (HPV) is the primary cause of cervical cancer and its precursors (5). Previous studies reported an increased risk for HPV-related cervical cancer in RTRs whose cell-mediated immunity is suppressed (6, 7). HPV is a small double-stranded DNA virus that belongs to Papillomaviridae, a large family with a tropism for squamous epithelial tissue. HPV is commonly divided into high-risk (hrHPV) or low-risk (lrHPV) groups based on its capacity to drive the development of cancer (8). HrHPV subtypes, i.e., HPV16, HPV18, HPV31, HPV33, HPV35, HPV39, HPV45, HPV51, HPV52, HPV56, HPV58, and HPV59 are evidently considered high risk, and HPV66 and HPV68 are less evidently high risk (9). These 14 hrHPV subtypes have been proven to cause more than 96% of cervical cancers (10). The hrHPV prevalence in RTRs varies in different areas. Although some studies reported no difference in hrHPV infection rates between kidney transplant recipients and their matched controls (11–13), most studies confirmed a significantly higher prevalence of cervical hrHPV among RTRs (14–17). The 2019 American Society of Colposcopy and Cervical Pathology (ASCCP) consensus guidelines emphasized the importance of regular cervical cancer screening for patients with immunosuppression (18). However, during the course of our investigation, we found low coverage of HPV screening programs in China, which means that cervical cancer remains a great threat to female RTRs. Compared to immunocompetent controls, detailed data on the genotype-specific prevalence of HPV, risk factors for HPV infection, and the morbidity of cervical lesions in RTRs are still unclear.

This retrospective study aimed to evaluate the genotype-specific prevalence of cervicovaginal hrHPV infection and the incidence of cervical lesions among Chinese RTRs and a group of healthy controls to identify the risk factors for cervicovaginal hrHPV infection in RTRs.

## Materials and methods

### Ethics statement

The Ethics Committee of the First Affiliated Hospital of Sun Yat-sen University approved this project. All of the samples and data were collected after written informed consent was provided by the participants. The management and publication of patient information in this research was strictly in accordance with the Declaration of Helsinki, including confidentiality and anonymity. No financial compensation was provided.

### Study population

The Department of Organ Transplant Center in the First Affiliated Hospital of Sun Yat-sen University is a tertiary referral center for patients with end-stage renal disease and one of the largest organ transplant centers in China. This study was designed as a single-center, retrospective study. From 2010 to 2020, patients receiving renal transplantation surgery (referred to as RTRs), who had been screened for HPV infection one year after transplantation were enrolled at the Department of Organ Transplant Center and the Department of Gynecology in the First Affiliated Hospital of Sun Yat-sen University, China. From the registration system of the medical examination center at the First Affiliated Hospital of Sun Yat-sen University, we randomly sampled a comparison cohort of four age- and marital status-matched healthy individuals for each RTR (referred to as non-RTRs).

The inclusion criteria were as follows: 1. age ≥18 years and ≤70 years; 2. received renal transplantation surgery during the period of Jan 1, 2010 to Apr 30, 2020; 3. therapy followed by an immunosuppression regimen over one year; 4. regular HPV detection after renal transplantation at an interval ≤ 3 years; 5. histologic and genotyping confirmation of HPV infection results obtained in our hospital; and 6. willingness to adhere to scheduled follow-up.

The exclusion criteria included kidney function loss (indicating that the use of immunosuppressive drugs had been reduced or stopped); prior uterine or vaginal surgery, HPV infection and HPV-related lesions before renal transplantation; more than once transplantation and incomplete cervical screening data.

Relevant clinical data about renal transplantation were all collected in the hospital database. Additional data were collected during follow-up: duration of HPV infection, sexual history, symptoms, location of the lesions, treatment of cervical lesions, time since organ transplantation, immunosuppression treatment protocol, and history of rejection. Samples for HPV genotyping and cytology of the patients were collected in the outpatient clinics of the Gynecology Department at the First Affiliated Hospital of Sun Yat-sen University. Samples of the healthy controls were collected in the medical examination center at our hospital. The diagnosis of cervical disease was confirmed *via* colposcopy and cervical biopsy in the outpatient clinics of the Gynecology Department at our hospital.

### Immunosuppressive therapy and co-medication

The patients were treated according to national guidelines and from 2010 according to the international Kidney Disease: Improving Global Outcomes (KDIGO) guidelines (19). Either CD25 monoclonal antibody (basiliximab) or anti-thymocyte globulin (ATG) was administered as induction therapy in kidney transplantations from living-related and cadaveric donors. Basiliximab was given at a dose of 20 mg on postoperative days 0 and 4. ATG was given at a dose of 50 mg during the transplant operation and daily in the following 2 days after transplant. Immunosuppressive maintenance therapy consisted of steroids, mycophenolate mofetil and a calcineurin inhibitor, mostly tacrolimus but alternatively cyclosporin. Acute T-cell-mediated rejection (TCMR) was treated with intravenous steroid pulse therapy. Antibody-mediated rejection (ABMR), combined (cellular and antibody-mediated) and steroid- resistant acute TCMR episodes were treated with plasma exchange, ATG, rituximab, and bortezomib.

### Sampling technique

#### Cytology detection

A cytobrush was used to sample the ecto/endocervical junction and inserted into a liquid-based cytology (LBC) vial (BD SurePath™, TriPath, Burlington, NC, USA) and transported daily, at room temperature to the laboratory. LBC samples were vortexed for 20 seconds and processed by the fully automated BD system. It uses 9 mL of SurePath1 liquid medium and performs “cell enrichment”, that is, the removal of possible interferences and the formation of epithelial cells pellet in the SlidePrep™ equipment. Following, the pre-processed material for oncotic cytology was

sent to the BD Totalys SlidePrep™ automated medium, for preparing and staining the slide. All cases were analyzed and reviewed by experienced cytologists. The results were reported according to the Bethesda System for reporting cervical cytology:

negative for intraepithelial lesion or malignancy; atypical squamous cells of undetermined significance (ASC-US); atypical squamous cells that cannot exclude high-grade squamous intraepithelial lesion (ASC-H); low-grade squamous intraepithelial lesion (LSIL) (encompassing: HPV/mild dysplasia/CIN 1) and high-grade squamous intraepithelial lesion (HSIL) (encompassing: moderate and severe dysplasia, cervical carcinoma *in situ* (CIS); CIN 2 and CIN 3); atypical glandular cells favor neoplastic and endocervical adenocarcinoma *in situ* (20).

#### DNA extraction and HPV genotyping

Cervical cells were collected with a cytobrush from ectocervix and endocervix of the uterus by cervical scrapings. The samples were stored at 4 °C in the standard media provided with the panel for DNA extraction. DNA isolation and purification were conducted according to the manufacturer’s instructions (Tellgen Corporation, Shanghai, China).

All HPV tests were performed with an HPV genotyping panel (polymerase chain reaction, PCR)-Luminex based assay (Tellgen Corporation, Shanghai, China), which identified 14 hrHPV types (16, 18, 31, 33, 35, 39, 45, 51, 52, 56, 58, 59, 66 and 68). HPV DNA was extracted, amplified, and genotyped according to the manufacturers’ protocol. The PCR program consisted of an initial step at 95 °C for 9 min, 40 cycles of 95 °C for 20 s, 55 °C for 30 s, 72 °C for 30 s, and a final extension at 72 °C for 5 min. Amplified PCR products are hybridized to sets of beads with coated HPV type-specific probes. After subsequent incubation with phycoerythrin (PE)-conjugated streptavidin (SA-PE), beads are read within a Luminex 200 system (Luminex Corporation, Texas). Sterile water and specimens with known HPV genotypes were used as the negative and positive controls, respectively.

### Questionnaire and follow-up

All participants were asked to complete a paper questionnaire. Questions included: 1. sociodemographic characteristics (i.e., educational level, religion and marital status); 2. medical data, regarding both past history and gynecological health; and 3. sexual behavior and sexual partners.

The distribution and analysis of the questionnaires were performed by five volunteers.

All participants with precursor lesions detected in this study were referred to a gynecologist at our hospital for treatment and follow-up.

### Statistical analysis

All data were pseudonymously stored in an electronic database for data collection and statistical analysis (IBM SPSS Statistics 20, New York). For continuous variables, medians (range) or means (±SD) were calculated, depending on the distribution of the parameters. For categorical variables, total numbers and percentages were calculated for each modality. The duration of immunosuppressive therapy was calculated as the time between date of transplantation and date of participation in the study. The modified Wald method was used to compute 95% confidence intervals (CI) for the HPV-prevalence and cytological abnormalities.

Logistic regression was used to estimate risk factors for HPV-infection among RTRs as odds ratios (OR) with 95% confidence intervals (CI). In the logistic regression analyses, a model including RTR characteristics as linear variables were constructed. Statistical significance was defined as a two-sided p-value < 0.05.

## Results

### Participants

**Figure 1:**
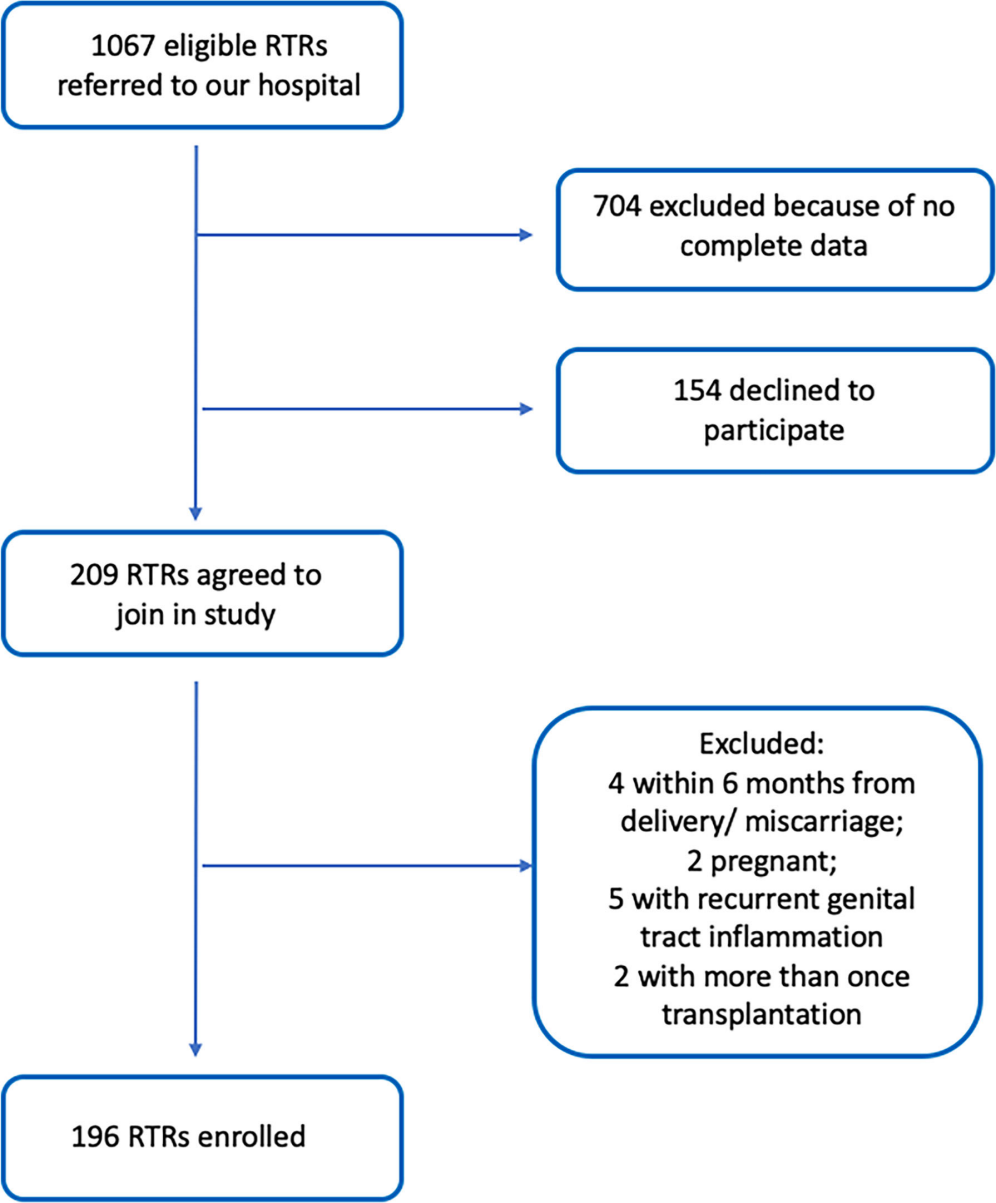
Flow diagram of RTR patient selection.

A total of 196 RTRs in the First Affiliated Hospital of Sun Yat-sen University from 2010 to 2020 were enrolled. The patient inclusion procedures are shown in **Figure 1**. Matched nontransplanted healthy individuals were selected from the medical examination center at our hospital. Thus, the final study population consisted of 196 RTRs and 784 non-RTRs.

**Table 1** shows the baseline characteristics of the study population. The median age of RTRs and non-RTRs at enrollment was 43.0 ± 9.3 years (range 23-70) and 42.8 ± 9.6 years old (range 23-71), respectively. The most common cause of renal transplantation was chronic glomerulonephritis (43.4%), followed by IgA nephropathy (20.4%). One patient received a combined pancreas and kidney transplant. There were no significant differences in age, educational level or marital status between the RTRs and non-RTRs. Since the coverage of HPV vaccination was very low, none of the RTRs had received HPV vaccination before baseline measurements. Meanwhile, only a small proportion of the control group had received HPV vaccination (1.8%).

**Table 1:** Baseline characteristics of Chinese renal transplant recipients (RTRs) and a comparison cohort (non-RTRs).

	RTR (n = 196)		Non-RTRs (n = 784)	
Age at inclusion	N	%	n	%
≤29	14	7.1	63	8.0
30-39	60	30.6	232	29.6
40-49	80	40.8	311	39.7
50-59	34	17.3	134	17.9
≥60	8	4.1	44	5.6
Educational level				
Basic	43	21.9	157	20.0
Medium	98	50.0	386	49.2
High	55	28.1	241	30.7
Marital status, n (%)				
Single	18	9.2	87	12.0
Married	172	87.8	630	80.4
Divorced	6	3.1	67	8.5
Cause of CKD, n (%)			NA	
Chronic glomerulonephritis	85	43.4		
IgA nephropathy	40	20.4		
Diabetic	8	4.1		
Vascular and hypertensive	4	2.0		
Lupus nephritis	5	2.6		
Other	11	5.6		
Unknown	43	30.0		
With other solid organ transplant, n (%)	1	0.5	NA	
With HPV vaccination, n (%)	0	0	14	1.8

HPV, human papillomavirus; RTR, renal transplant recipient; CKD, chronic kidney disease.

### The hrHPV prevalence and CIN2+ incidence rate were higher in RTRs than in non-RTRs

**Table 2** shows the hrHPV prevalence, abnormal cytology and cervical intraepithelial neoplasia occurrence in RTRs and the control group. The overall hrHPV infection and abnormal cytology rates in the RTR group were 23.5% (46/196) and 20.9% (41/196), higher than those in the control group (9.2% and 7.8%, respectively). The ORs of hrHPV infection and abnormal cytology in RTRs vs. non-RTRs were 3.033 (95% CI, 2.013-4.568) and 3.092 (95% CI, 2.007-4.764), respectively. Moreover, the occurrence of cervical intraepithelial neoplasia grade 2+ (CIN2+, including CIN2-3, CIS, AIS and Ca) confirmed by colposcopy biopsy was 10.2% (20/196) in RTRs, impressively higher than that in non-RTRs, with a rate of 2.2% (17/784). Female RTRs had an over 3 times higher hazard than non-RTRs during follow-up when matching at baseline for age, education, and marital status (OR = 3.628; 95% CI, 1.863-7.067, P<0.001).

**Table 2:** Odds ratio (OR) of HPV infection, abnormal cytology and cervical intraepithelial neoplasia in Chinese RTRs compared to those in a comparison cohort (non-RTRs).

	Group	Total	Events	OR	95%CI	*P* value
HPV infection						**<0.001**
	Non-RTRs	784	72	1		
	RTRs	196	46	3.033	2.013-4.568	
Abnormal cytology						**<0.001**
	Non-RTRs	784	61	1		
	RTRs	196	41	3.092	2.007-4.764	
CIN2+ lesion						**<0.001**
	Non-RTRs	784	17	1		
	RTRs	196	20	3.628	1.863-7.067	

HPV, human papillomavirus; RTR, renal transplant recipient; CIN, cervical intraepithelial neoplasia; OR, Odds ratio; CI, confidence interval. Bold values represent that the number is less than 0.05, which means there is a statistical difference.

### The HPV16 prevalence and multi-infection rate were more common in RTRs than in non-RTRs

**Figure 2:**
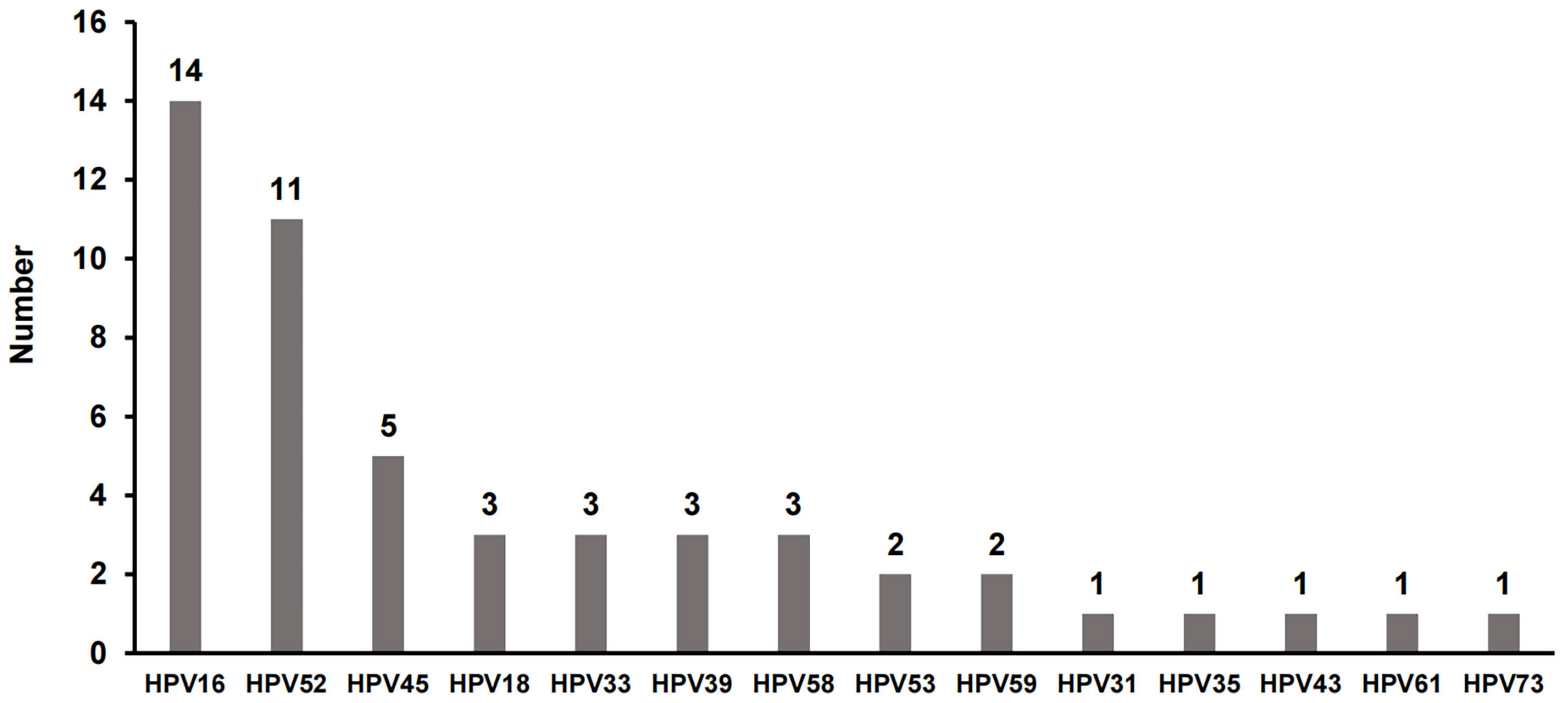
The distribution of high-risk HPV (hrHPV) genotypes in HPV-infected RTRs. There were 11 patients who had more than one type of hrHPV synchronously, including 2 cases with 3 types of HPV infection and 9 cases with two types of HPV infection.

Furthermore, hrHPV genotypes in RTRs were identified. The genotype distributions of hrHPV among RTRs are presented in **Figure 2**. In the female recipients, HPV16 was detected as the most prevalent genotype (30.4%), followed by HPV52 (23.9%), HPV45 (10.9%) and HPV18, 33, 39, 58 (6.5%). The multi-infection rate was high in HPV-infected RTRs, 23.9% (11/46). There were 2 cases with 3 types of HPV infection (16, 33, 35 and 18, 39, 73, respectively) and 9 cases with HPV coinfection (16 and 52 for 2 cases, 33 and 59 for 2 cases, 18 and 58, 39 and 52, 51 and 58, 52 and 58, 52 and 61 for the other 5 individual cases). The multi-infection rate was higher in RTRs (23.9% vs. 0.1%, P<0.001).

**Figure 3:**
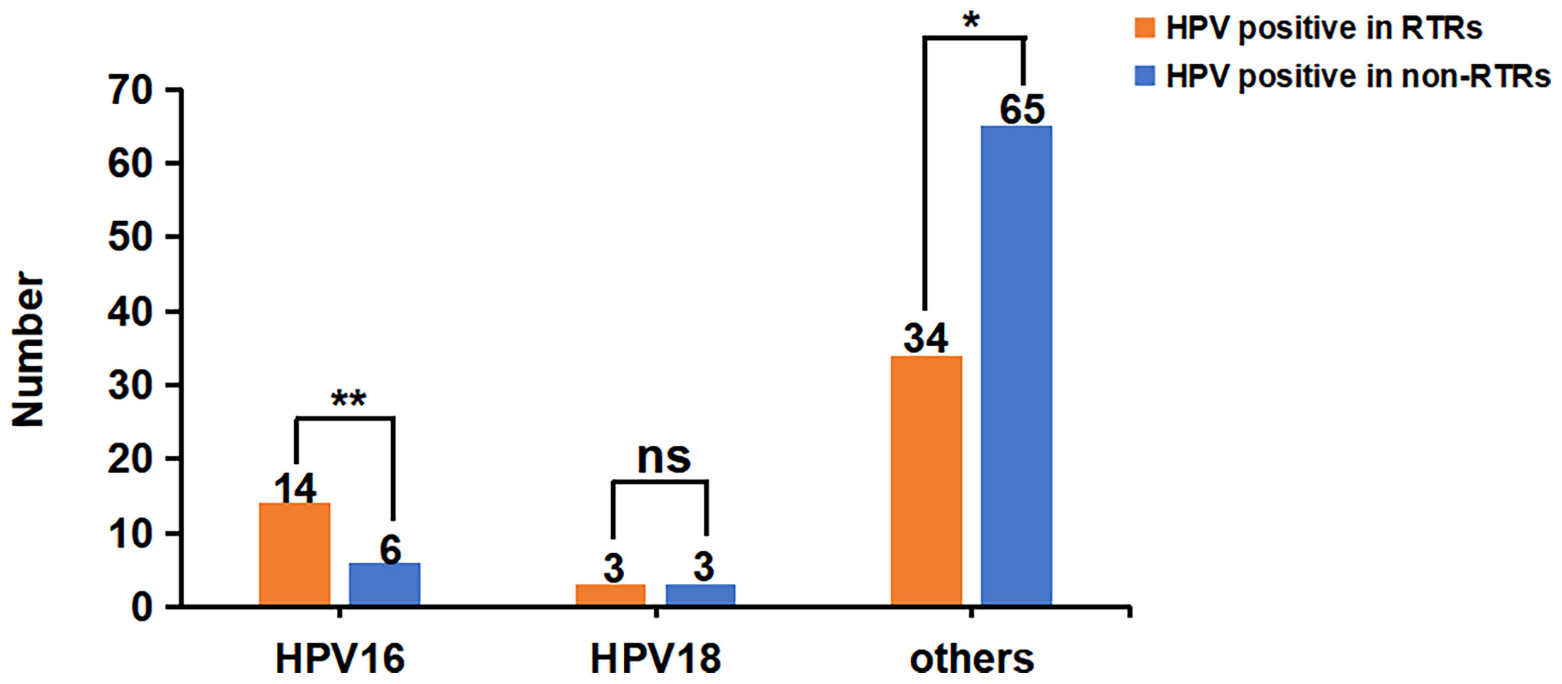
Comparison of different types of hrHPV infection in RTRs and non-RTRs. There were 46 RTRs and 72 non-RTRs with hrHPV infection, respectively. The prevalence of HPV16 in RTRs was much higher (30.4% vs. 8.3%, P=0.002), but the infection rate of HPV18 was not significantly different between the two groups (6.5% vs. 4.2%, P=0.890). The prevalence of hrHPV infection other than 16,18 in RTRs was lower than that in non-RTRs (73.9%vs. 90.3%, P=0.018). *P < 0.05; **P < 0.01; ns, no significance..

As shown in **Figure 3**, compared to the non-RTRs, the prevalence of HPV16 in RTRs was much higher (30.4% vs. 8.3%, P=0.002), while the prevalence of hrHPV infection other than 16,18 in RTRs was lower (73.9%vs. 90.3%, P=0.018).

We investigated the morbidity of CIN2+ disease in RTRs, as most cervical neoplasias were related to HPV. There were 20 cases with CIN2+ lesions, 25% of which were HPV coinfection (see supple **Table 1**). Among them, infection with HPV16 accounted for 50% of the severe cervical lesions (10/20). In the 10 cases of HPV 16 infection (including two cases of coinfection, with 18 and 52 infection, respectively), there were 9 patients with CIN3+ disease. All 4 cases of cervical squamous cancer were infected with HPV16. HPV 52 infection ranked second, leading to one CIN2, two CIN3 and one squamous cancer (coinfected with HPV16). And HPV 58 infection ranked third and caused two CIN2 and two CIN3, two of which were coinfected with 18 and 52 respectively. Other HPV types including 33, 45 and 59, mainly cause CIN2, and most of them are single infection. Interestingly, there was one case of CIN3 with HPV negative result.

### Clinical characteristics and risk factors for HPV infection among RTRs

As shown in **Table 3**, we compared multiple factors in the HPV-positive and HPV-negative groups in an attempt to identify the risk factors for HPV infection after kidney transplantation. A total of 176 out of 196 RTRs were on dialysis before transplantation. The average dialysis time of HPV-infected RTRs was 18.7 months, with no significant difference from that in non-HPV-infected RTRs (P=0.120). The obvious difference between the HPV-positive and HPV-negative groups was the months of immunosuppression (107.7 vs. 58.9 months, P<0.001). Five patients had acute immune rejection, accounting for 2.5% of all RTRs. No differences were found in smoking, diabetes, immunosuppressive regimens, acute rejection, cancer or infection history, number of pregnancies, childbirths or miscarriages.

**Table 3:** Univariate and multivariate analysis of risk factors for HPV infection in RTRs.

Characteristics	HPV positive (n=46)		HPV negative (n=150)	Univariable		Multivariable	
				OR (95%CI)	*P* value	OR (95%CI)	*P* value
Smoking							
No	46	148		1		–	–
Yes	0	2		1.014 (0.995-1.033)	1.000	–	–
Diabetes							
No	41	132		1	–	1	–
Yes	5	18		0.894 (0.313-2.558)	0.835	0.308 (0.063-1.504)	0.146
Age at inclusion							
≤29	2	12		1	–	1	–
30-39	13	47		1.660 (0.329-8.370)	0.539	0.842 (0.150-4.736)	0.845
40-49	18	62		1.742 (0.357-8.510)	0.439	0.587 (0.097-3.559)	0.562
50-59	9	25		2.160 (0.403-11.586)	0.369	0.976 (0.143-6.662)	0.980
≥60	4	4		6.000 (0.780-46.143)	0.085	3.749 (0.283-49.734)	0.316
Months of dialysis							
≤24	36	98		1	–	1	–
25-48	6	27		0.605 (0.231-1.586)	0.307	0.601 (0.204-1.774)	0.356
≥49	4	25		0.436 (0.142-1.338)	0.147	0.664 (0.183-2.410)	0.533
Current drugs							
Tacrolimus	39	128		0.958 (0.380-2.410)	0.927	–	–
Mycophenolate mofetil	16	58		0.846 (0.424-1.687)	0.635	–	–
Cyclosporine	7	20		1.167 (0.459-2.963)	0.746	–	–
Mycophenolate Sodium	26	68		1.568 (0.806-3.050)	0.186	–	–
Meprednisone	8	25		1.053 (0.439-2.525)	0.909	–	–
Prednisolone	30	110		0.682 (0.336-1.382)	0.288	–	–
Sirolimus	4	11		1.203 (0.364-3.977)	0.761	–	–
With other solid organ transplant							
No	46	149		1.000	–	–	–
Yes	0	1		1.309 (1.211-1.415)	1.000	–	–
With acute reject reaction							
No	44	147		1.000	–	1.000	–
Yes	2	3		2.227 (0.361-13.755)	0.389	1.299 (0.126-13.360)	0.826
Number of Pregnancies, median (SD)							
	1.93±1.451	1.62±1.587		1.130 (0.924-1.383)	0.234	1.201 (0.918-1.571)	0.181
Number of Childbirth, median (SD)							
	1.20±1.046	1.05±0.954		1.155 (0.833-1.601)	0.387	1.256 (0.804-1.962)	0.317
Number of Misbirth, median (SD)							
	0.74±1.104	0.57±1.161		1.123 (0.860-1.466)	0.396	1.162 (0.820-1.648)	0.399
History of cancer							
No	44	145		1.000	–	1.000	–
Yes	2	5		1.318 (0.247-7.032)	0.746	0.717 (0.091-5.630)	0.751
With infection of other virus							
BK Virus							
No	45	135		1.000	–	–	–
Yes	1	15		0.200 (0.026-1.557)	0.124	–	–
Hepatitis B Virus							
No	44	139		1.000	–	–	–
Yes	2	11		0.574 (0.123-2.691)	0.482	–	–
Cytomegalovirus							
No	46	149		1.000	1	–	–
Yes	0	3		1.309 (1.211-1.415)	1.000	–	–
Months of immunosuppression							
≤48	6	65		1.000	–	1.000	–
49-96	21	72		3.160 (1.201-8.312)	**0.020**	3.268 (1.180-9.049)	**0.023**
97-144	10	9		12.037 (3.522-41.138)	**<0.001**	14.714 (3.865-56.019)	**<0.001**
≥145	9	4		24.375 (5.749-103.342)	**<0.001**	33.663 (6.453-175.619)	**<0.001**

SD, standard deviation; RT, renal transplantation. Bold values represent that the number is less than 0.05, which means there is a statistical difference.

We further examined the associations between possible risk factors and hrHPV infection among RTRs. All factors were analyzed by univariate analysis, and some factors were also included in multivariate analysis based on clinical experience and other literatures. If a factor is subjected to both univariate analysis and multivariate analysis, the multivariate analysis is used as the final result. The age at inclusion, smoking history, diabetes history, months of dialysis, current drugs, other organ transplantation, acute rejection reaction, number of pregnancies, childbirths or miscarriages, infection of other virus and cancer history were not associated with hrHPV infection in RTRs. The only related factor of hrHPV infection was the duration of immunosuppression. The risk seemed to increase gradually with the time of immunosuppression (OR = 3.268 for immunosuppression of 49-96 months; OR = 14.714 for immunosuppression of 97-144 months and OR = 33.663 for immunosuppression over 144 months vs immunosuppression less than 48 months).

## Discussion

The main risk factor for cervical cancer is hrHPV infection, which presents a considerable threat of cervical cancer for female RTRs. However, available information on the incidence of cervical lesions in RTRs is scarce. This is the first study conducted in female Chinese RTRs to assess the incidence of HPV infection and posttransplant cervical lesions. In this study, we found that there were more cases of hrHPV infection and cervical premalignancies and cancers in female RTRs than in non-RTRs. The overall prevalence of cervicovaginal hrHPV infections in RTRs was 23.5%. HPV 16 was the most common genotype (30.4%) in RTRs. The only risk factor associated with an increased incidence of HPV infection in RTRs was the duration of immunosuppressive therapy. Our study demonstrates that Chinese RTRs have an elevated risk of CIN and cervical cancer.

The hrHPV prevalence in RTRs varies in different areas, and most studies found a significantly higher prevalence of cervical hrHPV among RTRs, ranging from 6.9% to 62.8% (14–17, 21, 22). Our cohort of female RTRs showed an elevated overall HPV prevalence of 23.5%, higher than the control group (9.2%), similar to most studies. The patients in our study had a limited mean number of sexual partners. Therefore, the most plausible explanation for the high HPV prevalence is delayed clearance and reactivation of latent infections due to the immunosuppressed state.

In addition, the markedly elevated HPV16 prevalence and multi-infection rate are characteristics of HPV infection in RTRs of our cohort. The genotypic spectrum of hrHPV infection among women varies worldwide. In Europe, HPV16, 18, 31, 33 and 58 are the most prevalent genotypes (23). HPV16, 52, 58, 53, and 18 are the top 5 subtypes with the highest infection rates in China (24). In our study, HPV16 was predominantly high in RTRs (30.4% vs. 8.3% in non-RTRs), followed by HPV52 (23.9%), HPV45 (10.9%) and HPV18, 33, 39, 58 (6.5%). Moreover, our data showed that the multi-infection rate was much higher in RTRs than in healthy controls (23.9% vs. 1.14%, P<0.001). Zhu reported that the prevalence of multiple HPV infections in the Chinese population was 3.44% (25). Overall, these results suggested that the prevalence of HPV16 and multiple infections is obviously related to immune status.

Nonetheless, the occurrence of CIN2+ lesions was impressively higher in RTRs than in non-RTRs (10.6% vs. 2.1%; P<0.001). Female RTRs had an over 3 times higher hazard of CIN2+ lesions than non-RTRs in our cohort during follow-up (OR=3.628;95% CI,1.863-7.067). According to Zhao’s pooled analysis that included 28761 cases, the age-standardized CIN2 prevalence was 1.5% (95% CI: 1.4-1.6%) and 0.7% (95% CI: 0.7-0.8%), and the CIN3+ prevalence was 1.2% (95% CI: 1.2-1.3%) and 0.6% (95% CI: 0.5-0.7%) in rural and urban Chinese women, respectively (26). Therefore, RTRs have a substantially higher risk of HPV-related genital premalignancies and cervical cancer than the general population. The possible reasons lie in the following: first, the high HPV16 infection rate increased RTRs’ risk of HPV-related disease, as HPV16 accounts for approximately 60% of all cervical cancers (27). In our 10 cases of HPV 16 infection (including two cases of coinfection), there were 9 patients with CIN3+ disease. All 4 cases of cervical squamous cancer were infected with HPV16. Second, patients with renal transplantation were reported to have a lower rate of spontaneous regression of abnormal cervical cytology (28). Abnormal cervical cytology in patients with organ transplantation tends to progress in a much shorter period of time than for nonimmunosuppressed patients. Therefore, cervical cancer screening of RTRs needs to be carefully monitored at a more regular interval (29).

We found that the only risk factor for HPV infection in RTRs was the duration of immunosuppressive treatment. The risk factors for genital HPV infection among RTRs have seldom been investigated and remain controversial due to different trial designs and sample sizes. According to the literatures, the relevant factors included the duration of immunosuppressive treatment, younger age, and a history of nulliparity (16, 30, 31). In contrast, some studies did not find any relationship between clinical data, such as immunosuppressive regimen, graft function, or time interval from transplantation, and hrHPV presence (11, 32). In our study, the risk of cervical HPV infection increased over time after transplantation. Because of reduced immune surveillance, RTRs have a disproportionate burden of persistent HPV infection and disease compared to the general population. The risk of HPV infection increased with time of the immunosuppressive therapy, which demonstrates that the majority of HPV-associated malignancies in RTRs are preventable when careful and routine Pap smear and HPV tests are performed after renal transplantation. We did not find other significant risk factors, including age, sexual behavior, number of sexual partners and marital status, related to HPV infection. The conservatism of Chinese women may be a reason. In our cohort, over 70% of patients had only one sexual partner (their husband) and had limited sexual activities after renal transplantation.

For the prevention of cervical cancer, prophylactic vaccines against hrHPV genotypes 16 and 18 are in high demand. It is important to evaluate the beneficial effect of this vaccine in transplant patients. Due to the lower immunogenicity of the quadrivalent HPV vaccine in transplant patients on immunosuppressive therapy, pretransplantation vaccination may be more effective (33). China has the largest domestic population, and the production of HPV vaccines and the vaccination process are in urgent need of improvement.

The limitation of this study is its retrospective nature at a single center. We hope to carry out a multicenter prospective study to evaluate the dynamics of HPV infections and cervical lesions in RTRs, which will provide more insight into the role of immunosuppression. Furthermore, the low coverage of HPV vaccines in China made the exploration of the effects of HPV vaccines in RTRs impossible.

In conclusion, RTRs had significantly higher HPV infection rates and increased risks of HPV-related cervical premalignancies and cancers due to their immunosuppressed state. The duration of immunosuppression is a risk factor for transplant recipients. These results support that female RTRs may benefit from more frequent cervical cancer screening after renal transplantation than healthy women. Prospective research on HPV infection dynamics in RTRs and optimal screening methods should be further explored in the future. The efficacy of vaccination in RTRs, both before and after transplantation, should be carefully assessed.

## Data availability statement

The original contributions presented in the study are included in the article/**Supplementary material**. Further inquiries can be directed to the corresponding authors.

## Author contributions

MiC: conceptualization, data curation, formal analysis, investigation, methodology, funding, and writing original draft. QC: data curation, formal analysis, investigation, methodology, and writing original draft. MeC: data curation, investigation, and methodology. MX: data curation, investigation, and methodology. DL: data curation, investigation, and methodology. PC: investigation and methodology. CW: conceptualization, data curation, and supervision. MH: conceptualization, investigation, and supervision. All authors contributed to the article and approved the submitted version.

## Funding

This study was funded by the National Natural Science Foundation of China 81602261and 81872118; CSCO Cancer Research Foundation (Y-sy2018-120); Beijing Kanghua Foundation (KH-2021-LLZX-052). The funders had no role in the study design, data collection and analysis, decision to publish, or preparation of the manuscript.

## Conflict of interest

The authors declare that the research was conducted in the absence of any commercial or financial relationships that could be construed as a potential conflict of interest.

## Publisher’s note

All claims expressed in this article are solely those of the authors and do not necessarily represent those of their affiliated organizations, or those of the publisher, the editors and the reviewers. Any product that may be evaluated in this article, or claim that may be made by its manufacturer, is not guaranteed or endorsed by the publisher.

## Supplementary material

The Supplementary Material for this article can be foundonline at: https://www.frontiersin.org/articles/10.3389/fonc.2022.905548/full#supplementary-material.

## References

[1] Johansen KL Chertow GM Foley RN Gilbertson DT Herzog CA Ishani A. US Renal data system 2020 annual data report: Epidemiology of kidney disease in the united states. Am J Kidney Dis (2021) 77(4 Suppl 1):A7–a8. doi: 10.1053/j.ajkd.2021.01.002 33752804 PMC8148988

[2] Zhang L Zhao MH Zuo L Wang Y Yu F Zhang H. China Kidney disease network (CK-NET) 2016 annual data report. Kidney Int Suppl (2011) (2020) 10(2):e97–e185. doi: 10.1016/j.kisu.2020.09.001 33304640 PMC7716083

[3] Bauer AC Franco RF Manfro RC. Immunosuppression in kidney transplantation: State of the art and current protocols. Curr Pharm Des (2020) 26(28):3440–50. doi: 10.2174/1381612826666200521142448 32436821

[4] Weikert BC Blumberg EA. Viral infection after renal transplantation: surveillance and management. Clin J Am Soc Nephrol (2008) 3 Suppl 2(Suppl 2):S76–86. doi: 10.2215/cjn.02900707 PMC3152274 18309006

[5] Steenbergen RD Snijders PJ Heideman DA Meijer CJ. Clinical implications of (epi)genetic changes in HPV-induced cervical precancerous lesions. Nat Rev Cancer (2014) 14(6):395–405. doi: 10.1038/nrc3728 24854082

[6] Chin-Hong PV Reid GE. Human papillomavirus infection in solid organ transplant recipients: Guidelines from the American society of transplantation infectious diseases community of practice. Clin Transpl (2019) 33(9):e13590. doi: 10.1111/ctr.13590 31077438

[7] Moscicki AB Flowers L Huchko MJ Long ME MacLaughlin KL Murphy J. Guidelines for cervical cancer screening in immunosuppressed women without HIV infection. J Low Genit Tract Dis (2019) 23(2):87–101. doi: 10.1097/lgt.0000000000000468 30907775

[8] Soto D Song C McLaughlin-Drubin ME. Epigenetic alterations in human papillomavirus-associated cancers. Viruses (2017) 9(9):248. doi: 10.3390/v9090248 PMC5618014 28862667

[9] Burd EM. Human papillomavirus laboratory testing: the changing paradigm. Clin Microbiol Rev (2016) 29(2):291–319. doi: 10.1128/cmr.00013-15 26912568 PMC4786885

[10] Arbyn M Tommasino M Depuydt C Dillner J. Are 20 human papillomavirus types causing cervical cancer? J Pathol (2014) 234(4):431–5. doi: 10.1002/path.4424 25124771

[11] Mazanowska N Pietrzak B Kamiński P Ekiel A Martirosian G Jabiry-Zieniewicz Z. Prevalence of cervical high-risk human papillomavirus infections in kidney graft recipients. Ann Transpl (2013) 18:656–60. doi: 10.12659/aot.884029 24300773

[12] Origoni M Stefani C Dell'Antonio G Carminati G Parma M Candiani M. Cervical human papillomavirus in transplanted Italian women: a long-term prospective follow-up study. J Clin Virol (2011) 51(4):250–4. doi: 10.1016/j.jcv.2011.05.017 21680237

[13] Pietrzak B Mazanowska N Ekiel AM Durlik M Martirosian G Wielgos M. Prevalence of high-risk human papillomavirus cervical infection in female kidney graft recipients: an observational study. Virol J (2012) 9:117. doi: 10.1186/1743-422x-9-117 22709394 PMC3489881

[14] de Oliveira Martins CA Do Val Guimarães ICC Velarde LGC. Relationship between the risk factors for human papillomavirus infection and lower genital tract precursor lesion and cancer development in female transplant recipients. Transpl Infect Dis (2017) 19(4). doi: 10.1111/tid.12714 28456141

[15] Meeuwis KA Hilbrands LB IntHout J Slangen BF Hendriks IM Hinten F. Cervicovaginal HPV infection in female renal transplant recipients: an observational, self-sampling based, cohort study. Am J Transpl (2015) 15(3):723–33. doi: 10.1111/ajt.13053 25675976

[16] Parra-Avila I Jimenez-Santana ML Barron-Sanchez RE Martinez-Gamboa RA Alberu J Morales-Buenrostro LE. Incidence of cervical intraepithelial lesions and human papilloma virus infection in female renal transplant recipients. Transpl Infect Dis (2021) 23(4):e13622. doi: 10.1111/tid.13622 33877726

[17] Wielgos A Pietrzak B Sikora M Martirosian G Suchonska B Gozdowska J. Human papillomavirus (HPV) DNA detection using self-sampling devices in women undergoing long term immunosuppressive therapy. Viruses (2020) 12(9):962. doi: 10.3390/v12090962 PMC7552011 32872666

[18] Perkins RB Guido RS Castle PE Chelmow D Einstein MH Garcia F. 2019 ASCCP risk-based management consensus guidelines for abnormal cervical cancer screening tests and cancer precursors. J Low Genit Tract Dis (2020) 24(2):102–31. doi: 10.1097/lgt.0000000000000525 PMC7147428 32243307

[19] Kasiske BL Zeier MG Chapman JR Craig JC Ekberg H Garvey CA. KDIGO clinical practice guideline for the care of kidney transplant recipients: a summary. Kidney Int (2010) 77(4):299–311. doi: 10.1038/ki.2009.377 19847156

[20] Nayar R Wilbur DC. The Pap Test and Bethesda 2014. "The reports of my demise have been greatly exaggerated." (after a quotation from Mark Twain). Acta Cytol (2015) 59(2):121–32. doi: 10.1159/000381842 25997404

[21] Ghazizadeh S Lessanpezeshki M Nahayati MA. Human papilloma virus infection in female kidney transplant recipients. Saudi J Kidney Dis Transpl (2011) 22(3):433–6. PMID: 21566296 21566296

[22] Veroux M Corona D Scalia G Garozzo V Gagliano M Giuffrida G. Surveillance of human papilloma virus infection and cervical cancer in kidney transplant recipients: preliminary data. Transplant Proc (2009) 41(4):1191–4. doi: 10.1016/j.transproceed.2009.03.015 19460514

[23] WHO/ICO Information Centre on HPV and Cervical Cancer. HPV and cervical cancer in the 2007 report. Vaccine (2007) 25 Suppl 3:C1–230. doi: 10.1016/s0264-410x(07)01183-8 18068032

[24] Li K Li Q Song L Wang D Yin R. The distribution and prevalence of human papillomavirus in women in mainland China. Cancer (2019) 125(7):1030–7. doi: 10.1002/cncr.32003 30748006

[25] Zhu B Liu Y Zuo T Cui X Li M Zhang J. The prevalence, trends, and geographical distribution of human papillomavirus infection in China: The pooled analysis of 1.7 million women. Cancer Med (2019) 8(11):5373–85. doi: 10.1002/cam4.2017 PMC6718589 31350872

[26] Zhao P Liu S Zhong Z Hou J Lin L Weng R. Prevalence and genotype distribution of human papillomavirus infection among women in northeastern guangdong province of China. BMC Infect Dis (2018) 18(1):204. doi: 10.1186/s12879-018-3105-x 29724192 PMC5934871

[27] Crosbie EJ Einstein MH Franceschi S Kitchener HC. Human papillomavirus and cervical cancer. Lancet (2013) 382(9895):889–99. doi: 10.1016/s0140-6736(13)60022-7 23618600

[28] Tanaka Y Ueda Y Kakuda M Kubota S Matsuzaki S Nakagawa S. Clinical outcomes of abnormal cervical cytology and human papillomavirus-related lesions in patients with organ transplantation: 11-year experience at a single institution. Int J Clin Oncol (2016) 21(4):730–4. doi: 10.1007/s10147-015-0940-2 26694812

[29] Courtney AE Leonard N O'Neill CJ McNamee PT Maxwell AP. The uptake of cervical cancer screening by renal transplant recipients. Nephrol Dial Transpl (2009) 24(2):647–52. doi: 10.1093/ndt/gfn607 18952575

[30] Reinholdt K Thomsen LT Dehlendorff C Larsen HK Sorensen SS Haedersdal M. Human papillomavirus-related anogenital premalignancies and cancer in renal transplant recipients: A Danish nationwide, registry-based cohort study. Int J Cancer (2020) 146(9):2413–22. doi: 10.1002/ijc.32565 31291470

[31] Rose B Wilkins D Li W Tran N Thompson C Cossart Y. Human papillomavirus in the oral cavity of patients with and without renal transplantation. Transplantation (2006) 82(4):570–3. doi: 10.1097/01.tp.0000231706.79165.e5 16926603

[32] Webster AC Craig JC Simpson JM Jones MP Chapman JR. Identifying high risk groups and quantifying absolute risk of cancer after kidney transplantation: a cohort study of 15,183 recipients. Am J Transpl (2007) 7(9):2140–51. doi: 10.1111/j.1600-6143.2007.01908.x 17640312

[33] Kumar D Unger ER Panicker G Medvedev P Wilson L Humar A. Immunogenicity of quadrivalent human papillomavirus vaccine in organ transplant recipients. Am J Transpl (2013) 13(9):2411–7. doi: 10.1111/ajt.12329 PMC4583130 23837399

