# Supplementary material for: Risk of human papillomavirus infection and cervical intraepithelial lesions in Chinese renal transplant recipients
Source: Front Oncol. 2022 Jul 25;12:905548. doi: 10.3389/fonc.2022.905548 (PMC9359460; doi:10.3389/fonc.2022.905548)
Supplement: Supplementary file 1 [file Table_1.docx]

Suppl Table 1.  The distribution of high-risk HPV genotypes in RTRs developing cervical intraepithelial neoplasia grade 2+ lesions

| HPV genotype | CIN2 | CIN3 | CIS | Squamous Cancer |
| --- | --- | --- | --- | --- |
| 16 | 1 | 3 | 1 | 3 |
| 33 | 1 |  |  |  |
| 45 | 1 |  |  |  |
| 52 | 1 | 2 |  |  |
| 58 | 1 | 1 |  |  |
| 16、18 |  | 1 |  |  |
| 16、52 |  |  |  | 1 |
| 18、58 |  | 1 |  |  |
| 33、59 | 1 |  |  |  |
| 52、58 | 1 |  |  |  |
| HPV negative |  | 1 |  |  |

Abbreviation: CIN, cervical intraepithelial neoplasia；CIS, cervical carcinoma in situ.
